# Modified Monosaccharides Content of Xanthan Gum Impairs Citrus Canker Disease by Affecting the Epiphytic Lifestyle of *Xanthomonas citri* subsp. *citri*

**DOI:** 10.3390/microorganisms9061176

**Published:** 2021-05-29

**Authors:** Simone Cristina Picchi, Laís Moreira Granato, Maria Júlia Festa Franzini, Maxuel Oliveira Andrade, Marco Aurélio Takita, Marcos Antonio Machado, Alessandra Alves de Souza

**Affiliations:** 1 Biotechnology Lab, Centro de Citricultura Sylvio Moreira, Instituto Agronômico de Campinas, Cordeirópolis, São Paulo 1349070, Brazil; scpicchi@gmail.com (S.C.P.); mjuliafranzini@hotmail.com (M.J.F.F.); marco.takita@ccsm.br (M.A.T.); marcos@ccsm.br (M.A.M.); 2 Bioscience National Lab, Centro Nacional de Pesquisa em Energia e Materiais, Campinas, São Paulo 13083100, Brazil; maxuel_andrade@hotmail.com

**Keywords:** *X. citri*, phosphoglucomutase, EPS, saccharides chain, biofilm, motility

## Abstract

*Xanthomonas citri* subsp. *citri* (*X. citri*) is a plant pathogenic bacterium causing citrus canker disease. The *xanA* gene encodes a phosphoglucomutase/phosphomannomutase protein that is a key enzyme required for the synthesis of lipopolysaccharides and exopolysaccharides in Xanthomonads. In this work, firstly we isolated a *xanA* transposon mutant (*xanA*::Tn5) and analyzed its phenotypes as biofilm formation, xanthan gum production, and pathogenesis on the sweet orange host. Moreover, to confirm the *xanA* role in the impaired phenotypes we further produced a non-polar deletion mutant (Δ*xanA*) and performed the complementation of both *xanA* mutants. In addition, we analyzed the percentages of the xanthan gum monosaccharides produced by *X. citri* wild-type and *xanA* mutant. The mutant strain had higher ratios of mannose, galactose, and xylose and lower ratios of rhamnose, glucuronic acid, and glucose than the wild-type strain. Such changes in the saccharide composition led to the reduction of xanthan yield in the *xanA* deficient strain, affecting also other important features in *X. citri*, such as biofilm formation and sliding motility. Moreover, we showed that *xanA*::Tn5 caused no symptoms on host leaves after spraying, a method that mimetics the natural infection condition. These results suggest that *xanA* plays an important role in the epiphytical stage on the leaves that is essential for the successful interaction with the host, including adaptive advantage for bacterial *X. citri* survival and host invasion, which culminates in pathogenicity.

## 1. Introduction

*Xanthomonas citri* subsp. *citri* (*X. citri*) is a Gram-negative plant pathogenic bacterium causing citrus canker, one of the most economically damaging diseases that affects almost all commercial citrus varieties worldwide [1,2]. *X. citri* invades the host through natural openings, such as stomata and wounds [2], and many studies have shown that biofilms formed on the leaf surface and bacterial motility are important features in the early stages of infection [3,4]. Notably, mutants of *X. citri* impaired in biofilm formation, and/ or motility results in the decrease of citrus canker symptoms [3,4,5,6,7,8,9]. Additionally, either biofilm or sliding motility is affected by *X. citri* ability to produce exopolysaccharides (EPS) [4]. Therefore, EPS production in *X. citri* interferes directly with citrus canker disease development.

The main EPS produced by Xanthomonads is the xanthan, which is a polymer of repeating pentasaccharide units with the mannose-(β-1,4)- glucuronic acid-(β-1,2)-mannose-(α-1,3)-cellobiose structure [10,11]. Production of xanthan involves many genes, including the *gum* gene cluster, composed of twelve genes (*gumBCDEFGHIJKLM*), and the *xanA* gene (XAC3579), that encodes a phosphoglucomutase/phosphomannomutase (PGM/PMM) homologous [11,12]. XanA is a conserved enzyme in the Xanthomonadaceae family that provides essential 1-phosphosugars required for the biosynthesis of lipopolysaccharide and exopolysaccharide [11,13]. Genetic and biochemical analysis showed that *X. campestris* XanA is involved in the biosynthesis of both glucose 1-phosphate and mannose 1-phosphate and that a single mutation in *xanA* led to a drastic decrease of enzyme activity, though low levels of the activity remained detectable [11]. Furthermore, colonies of the *X. citri xanA* mutant were less viscous/sticky than the wild type and did not show PGM activity [12], besides demonstrated late canker symptoms on infiltrated and detached *Citrus aurantifolia* leaves [12].

Although a previous study in *X. citri* indicated that the PGM activity of XanA may be involved with xanthan synthesis, xanthan gum content and yield was not analyzed. Furthermore, the method used to verify bacterial pathogenicity did not evaluate the leaf epiphytic colonization, since *X. citri* survive on leaf surface forming biofilm before invading the parenchyma tissue. Indeed, the epiphytical stage has an important role in the *X. citri* pathogenesis [3] and is positively affected by xanthan gum production, which is crucial to pathogen survival and interaction with the host [4].

In this work, we expanded the investigation of *xanA* function, studying two different *xanA* mutants, to verify the xanthan gum composition and yield, besides to analyze important lifestyle traits of *X. citri*, as biofilm formation and motility. Further, we used a method that mimics the natural process of infection by *X. citri* to test the bacterial pathogenicity on *Citrus sinensis* leaves. Further, we showed changes in the xanthan gum monosaccharide composition that may be involved with the impairment of pathogenicity, which gives insight into the development of a targeted strategy to reduce citrus canker disease.

## 2. Materials and Methods

### 2.1. Screening Of Mutants and Identification of Transposon Insertion Sites

The screening for the biofilm formation-affected mutants in a mutant library of *X. citri* strain 306 (National Center for Biotechnology Information, NCBI, accession No: AE008923), constructed previously [14] using the EZ::TN < KAN-2 > transposome complex (Epicentre Technologies, Madison, WI) was performed using a polystyrene 96-well plate (Nunclon surface, Nuncbrand, Roskilde, Denmark) assay system [8]. After that, was selected a clone, identified as A7 that showed a decrease in biofilm formation.

For mapping the location of transposon insertion, thermal asymmetric interlaced PCR (TAIL-PCR) was performed to amplify unknown DNA sequences contiguous to known kanamycin gene sequences, according to [15]. Three successive high- and low-stringency PCR amplifications were performed with nested sequence-specific primers with a melting temperature (Tm) > 65 °C in consecutive reactions together with a short (15–16 nucleotides) arbitrary degenerate (AD1) primer with a Tm of about 45° C, and genomic templates from the mutant strain generated by random insertion. TAIL-PCR reactions were analyzed on a 1.0% agarose TBE gel and the brightest band was purified with a PCR Purification Kit (Wizard^®^ SV Gel and PCR Clean-Up System, Promega). The insertion point of transposon mutant was confirmed by DNA sequencing with the forward and reverse primers of EZ::TN (Epicentre Technologies, Madison, WI, USA) (Table 1). Sequences were compared and aligned with sequences from the GenBank database, by using the BLAST program of the National Center for Biotechnology Information (NCBI) website (http://www.ncbi.nlm.nih.gov/, access date: 27 January 2015). To verify the accuracy of transposon insertions in the sequence, PCR amplification was performed using primers (XAC3579F and XAC3579R) cited in Table 2, designed from the sequences flanking the gene XAC3579 (*xanA*). The PCR amplification was carried out in 1 cycle at 94 °C for 2 min, 35 cycles at 94 °C for 30 s, 56 °C for 30 s and 72 °C for 4 min, and 1 cycle at 72 °C for 10 min.

### 2.2. Bacterial Strains and Growth Conditions

*Xanthomonas citri* subsp. *citri* strain 306 (ampicillin-resistant) [16] and mutant strains were grown at 28 °C in NBY nutrient medium (0.5% (*w*/*v*) peptone, 0.3% (*w*/*v*) meat extract, 0.2% (*w*/*v*) yeast extract, 0.2% (*w*/*v*) K_2_HPO_4_, 0.05% (*w*/*v*) KH_2_PO_4_, pH 7.2) shaking at 180 rpm overnight or on 1.2% (*w*/*v*) agar solid media by 48 h. When required, antibiotics were added at the following concentrations: ampicillin (Ap) 100 μg/mL, kanamycin (Km) 50 μg/mL, gentamycin (Gm) 5 μg/mL. *Escherichia coli* DH5α cells were grown at 37 °C in Luria-Bertani (LB) medium (1% (*w*/*v*) tryptone, 0.5% (*w*/*v*) yeast extract and 1% (*w*/*v*) sodium chloride, pH 7.5) shaking at 200 rpm or on plates. The bacterial strains and plasmids used in this study are listed in Table 2.

Bacterial growth in 96-well microtiter plates was measured in the Varioskan Flash Multimode Reader (Thermo Fisher Scientific) at 600 nm (OD600).

### 2.3. Construction of the XanA Deletion Mutant and Complementation of EZ-Tn5 Insertion and Deletion Mutants of the X. Citri

Bacterial genomic DNA and plasmid DNA were extracted using a Wizard genomic DNA purification kit and a Wizard miniprep DNA purification system according to the manufacturer’s instructions (Promega, Madison, WI, USA). The concentration and purity of the DNA were determined using a Nanodrop ND-1000 spectrophotometer (NanoDrop Technologies, Wilmington, DE, USA). DNA was stored in Tris-EDTA buffer (10 mM Tris, 1 mM EDTA, pH 8.0) at −20 °C. PCR was performed using standard procedures [20] with Pfu DNA polymerase (Promega Corporation, Madison, WI, USA). The restriction digestions and DNA ligations were performed according to the manufacturer’s instructions.

To construct the *xanA* deletion mutant, approximately 1 kbp of the upstream and downstream regions of the *xanA* gene (XAC3579) was amplified using PCR from genomic DNA obtained from *X. citri* strain 306 using the following primer pairs: xanAF1 (5′-GGCTGGCGCCAAGCTTCCGACTGCAGCCACACATCGA-3′) and xanAR1 (5′-CAATCAGGCGGGTAGCGTCATGGGCAAATCCTG-3′); and xanAF2 (5′-TACCCGCCTGATTGACCCCTCTCCCACCCATAGAC-3′) and xanAR2 (5′-TCCTGCAGAGAAGCTTGGTGTTCTGGCAATCGAGCTGGATCAC-3′), respectively. Fragments were amplified using a high-fidelity polymerase (Phusion, Thermo Scientific) and PCR reaction conditions were: 98 °C for 4 min, 35 amplification cycles were performed at 95 °C for 1 min, 60 °C for 30 s, 72 °C for 1 min, and final incubation at 72 °C for 5 min. The PCR products were digested with *BamH*I, and both fragments were ligated to produce a deletion construct fragment containing 2 kbp. Thus, the resulting fragment was cloned into the pNPTS138 suicide vector to generate pNPTS_xanA (Table 2) using the *Hind*III restriction site, as previously described in Andrade et al. (2014) [21]. The pNPTS_xanA construction was introduced into *E. coli* by heat-shock at 42 °C [20] according to standard procedures and into *X. citri* by electroporation [22]. *X. citri* transformants were grown in LB medium plus kanamycin for 3 days at 28 °C and after the second recombination event *X. citri* cells were plated on LB medium containing ampicillin and 5% sucrose [23]. *X. citri* recombinant cells were grown for 2 days at 28 °C and after confirmation of the loss of both the kanamycin resistance cassette and *sacB gene*, a PCR was performed using the primers xanAF1 and xanAR2 mentioned above. *X. citri* strains carrying the *xanA* mutant allele were selected for further studies.

To complement the *xanA* EZ-Tn5 insertion and deletion mutants, a 1300 bp DNA fragment containing the entire *xanA* gene was amplified by PCR using total DNA obtained from the *X. citri* wild-type strain 306 as the template and the specific primer pair xanA_p53_F (5′ATTATTGGTACCATGACGCTACCCGCCTTCAAG3′) and xanA_p53_R (5′ATTATTAAGCTTTCAGCCGCGCAGCAGGTTAGA3′). The amplified DNA fragment was cloned into pUFR053 [19] at the *BamH*I and *Hind*III restriction sites to obtain the recombinant plasmid pUFR053_xanA (Table 2), which was used for genetic complementation. The construction was confirmed using sequencing. The recombinant plasmid pUFR053_xanA was transferred into *xanA*::Tn5 and Δ*xanA* mutant strains using electroporation, and cells were selected on NBY solid media using gentamicin, resulting in the strain *xanA*::Tn5_c and Δ*xanA*_c (xanA+).

### 2.4. Biofilm Formation on Abiotic Surfaces

Biofilms that formed on glasses and polystyrene plates were examined as previously described [3] with some modifications. Wild-type, mutants (*xanA*::Tn5 and Δ*xanA*), and the complemented (*xanA*::Tn5_c and Δ*xanA*_c) strains were grown into NBY overnight and the bacteria were centrifuged for six minutes at 4456 g. The optical density (OD) at 600 nm was adjusted to 0.1 (10^8^ CFU/mL) using fresh NBY plus glucose (1% *w*/*v*). The bacterial suspension was transferred into each glass tube or polystyrene plate and incubated at 28 °C without shaking for 48 h. Culture media were removed and bacterial cells attached to the abiotic surfaces were gently washed three times with sterilized distilled water and stained with 2 mL 0.1% Crystal Violet (CV) for 30 min. The unbound CV was poured off, and the surfaces were washed with water. The CV-stained cells were solubilized in 2 mL of ethanol. The samples were measured at 590 nm using a spectrometer (UV/Vis Spectrometer Lambda Bio; Perkin Elmer). Biofilm values were normalized to bacterial growth (OD at 600 nm). Assays were repeated two times independently with six replicates each.

### 2.5. Xanthan Gum Quantification

Bacteria were grown overnight at 28 °C with shaking at 180 rpm in NBY medium. Bacteria were adjusted to 0.1 (10^8^ CFU/mL) and 5 mL was transferred to 45 mL of NBY plus glucose (2% *w*/*v*). After 48 h of growth at 28 °C and shaking at 180 rpm, the cells were pelleted by centrifugation (4456 g for 6 min), and 3 volumes of 100% ethanol were added to the supernatant. The crude xanthan was collected using a glass rod and placed on a Petri dish to dry at 60 °C for 24 h. The assay was repeated three times independently with three replicates for each one.

### 2.6. Bacterial Motility Assay

Assays were performed as previously described [3,5]. Briefly, bacteria were grown overnight in NBY medium, and 3 µL of bacterial culture (10^6^ CFU/mL) was then plated onto SB medium plus 0.5% (w/v) agar for sliding analysis [24]. Plates were incubated at 28 °C for 48 h. Motility analysis was assessed qualitatively by examining the circular halo formed by the bacterial growth. The assay was performed in triplicate and repeated two times independently. The diameters of the circular halos that were occupied by each strain were measured, and the resulting values were taken to indicate the motility of *X. citri* strains. The assay was repeated three times independently with three replicates for each one.

### 2.7. Pathogenicity Assay

Pathogenicity assays were performed by three different methods. *X. citri* strains (*X. citri* wild-type and *xanA* EZ-Tn5 insertion mutant) that were grown in selective antibiotic NBY medium overnight at 28 °C, centrifuged at 4456× *g*, and then suspended in a 10 mM potassium phosphate buffer (pH 7.0).

Leaves from sweet orange (*Citrus sinensis* cv. ‘Bahia’) plants were inoculated by spraying and infiltration. In the first method, the abaxial surfaces of fully expanded immature leaves of each plant were sprayed with 10^8^ CFU/mL from each *X. citri* strain. In the second method, leaves were inoculated by pressure infiltration of 100 uL of bacterial suspension (10^4^ CFU/mL) from each *X. citri* strain. Phosphate buffer was used as the control in non-infected plants. All plant inoculations involved a minimum of three immature leaves from each plant, and three plants were inoculated for each bacterial strain. The plants were kept in the greenhouse at Instituto Agronômico de Campinas/IAC (Campinas, SP), at a temperature of 28 ± 4 °C in high humidity for 30 days. Disease symptoms were evaluated until 30 days post-inoculation, and assays were independently repeated twice.

In the third method, sweet orange leaves were pierced with a microneedle device (0.5 mm). Care was taken to apply gentle pressure onto the leaf abaxial surface with one move horizontally over the area. Bacteria strains were inoculated using a cotton pad soaked with the different *X. citri* strains (10^8^ CFU/mL). Three plants were inoculated for each *X. citri* strain and five leaves of each plant were used. The plants were kept at a temperature of 28 ± 4 °C and disease symptoms were evaluated after 15 days post-inoculation (dpi). The assay was repeated two times independently.

### 2.8. Xanthan Gum Composition Assessment in XanA Mutant and Wild-Type

Xanthan gum produced by wt and *xanA*::Tn5 mutant were evaluated by high-performance liquid chromatography (HPLC) according to the pre-established method [25] at “Laboratório de Bioquímica de Plantas” of UNESP in Jaboticabal (SP/Brazil). First of all, bacteria were grown for 72 h in NBY medium plus glucose (1% *w*/*v*) at 28°C and 200 rpm. The cells were pelleted by centrifugation (4456 g for 6 min) and 3 volumes of 100% ethanol were added to the supernatant. The crude xanthan was collected using a glass rod. A blend of three samples, totalizing 1 mg xanthan gum, was hydrolyzed with trifluoracetic acid (4 mol/L) and the monosaccharides were marked with the addition of 1-fenil-3-metil-5-pirazolone (PMP) solution (0.5 mol/L and methanol) and sodium hydroxide solution (0.3 mol/L). The microtubes were vortexed and incubated at 70 °C for 2 h. After cooling, the mixture was neutralized by a hydrochloric acid (0.3 mol/L) addition. The monosaccharides extraction was performed by the addition of butyl ether. The organic phase (supernatant) was removed by centrifugation at 5000 g for 5 min and one mL of milli-Q water was added in the remaining phase. The monosaccharides detection was performed at 245 nm by HPLC equipped with UV/VIS detector (Shimadzu, model SPD-M10A) using column GHRC ODS-C-18 (4.6 mm i.d. × 15 cm), flow rate of 0.5 mL/min, buffers A (ammonium acetate 100 mmol/L, pH 5.5 + 10% (*w*/*v*) acetonitrile) and B (ammonium acetate 100 mmol/L, pH 5.5 + 25% (*w*/*v*) acetonitrile) and separation gradient of 0% (30 min) and 0–100% up 100 min. Pattern monosaccharides were used for standard curve construction in the following concentrations: 0.0125, 0.0250, 0.0500, 0.1000 mg/mL.

### 2.9. Statistical Analysis

Data from biofilm formation, sliding motility, gum production, and pathogenicity assays were statistically analyzed using a t-test (*p* < 0.05). The values were expressed as the means ± standard deviations of independent replicates.

## 3. Results

### 3.1. XanA Encodes a Phosphoglucomutase Protein, Important to the Biofilm Formation and Xanthan Gum Production

**Figure 1:**
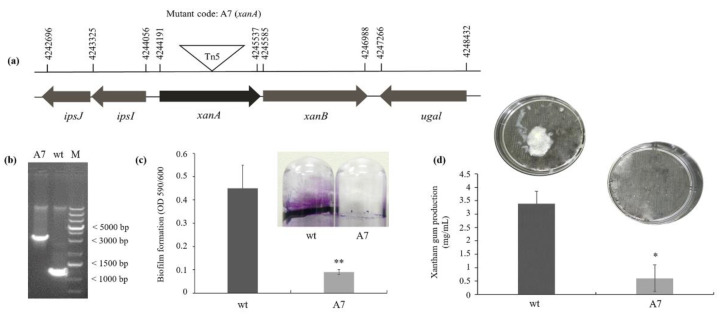
Identification of the A7 mutant from an EZ-Tn5 library of *X. citri* by biofilm and xanthan gum assays. (**a**) Genetic organization of the *xanA* gene in the *X. citri* subsp. *citri* strain 306 genome and the lengths of the open reading and transposon insertion site in the *xanA* mutant are indicated. The length of each arrow represents the relative open reading frame (ORF) size and indicates the direction of transcription. The triangle indicates the Tn5 insertion site. The annotation information and sizes of the genes were obtained from the genome sequence database of *X. citri* strain 306 (National Center for Biotechnology Information, NCBI, accession No: AE008923). (**b**) PCR analysis confirmed the insertion of EZ-Tn5 in the *xanA* gene (XAC_RS18095 or XAC3579). DNA was amplified using the primers XAC3579F and XAC3579R targeting a 500-bp region surrounding *xanA* from the wild-type (wt) and A7 (*xanA*::Tn5) strains, demonstrating the increase in the length of the obtained PCR amplicon by insertion of EZ-Tn5 transposon (1.22 kb) generating a PCR product with 2.22 kb in A7 mutant. M: Thermo Scientific O’GeneRuler 1 kb Plus DNA Ladder. (**c**) Biofilm formation on the abiotic surface by the wt and A7 mutant strains. The values were normalized to bacterial growth (OD at 600 nm). Values are expressed as the means ± standard deviations of six biological replicates. (**d**) Xanthan gum production. Values are expressed as the means ± standard deviations of three biological replicates. * indicates significant difference by t-test (*p* < 0.05) compared with wild-type.

The A7 mutant was isolated from an EZ-Tn5 transposon mutagenesis library of *X. citri* strain 306 [5,14] since it exhibited a reduced ability to form biofilm. Sequencing indicated that EZ-Tn5 was inserted in the position 651 downstream of the translation start site of the locus XAC_RS18095 (Figure 1a). PCR using the primers XAC3579F and XAC3579R demonstrated the increase in the length of the obtained PCR amplicon by insertion of EZ-Tn5 transposon (1.22 kb) generating a PCR product with 2.22 kb, which confirmed the mutation of the *xanA* gene (locus XAC_RS18095) (Figure 1b). Thus, we will refer to the A7 mutant as *xanA*::Tn5. The *xanA* gene encodes a predicted phosphoglucomutase protein, required to synthesize the xanthan gum, a pathogenesis-related exopolysaccharide in Xanthomonads [13].

The *xanA*::Tn5 produced approximately 80% less biofilm compared with the *X. citri* wild-type (wt) strain (Figure 1c). We further verified the importance of the mutated gene in xanthan gum production. As summarized in Figure 1d, the xanthan gum yield of *xanA*::Tn5 was significantly different from that of the wild-type strain, showing 83% less xanthan gum production.

### 3.2. Mutant Strain XanA::Tn5 Developed No Symptoms on Sweet Orange Host

**Figure 2:**
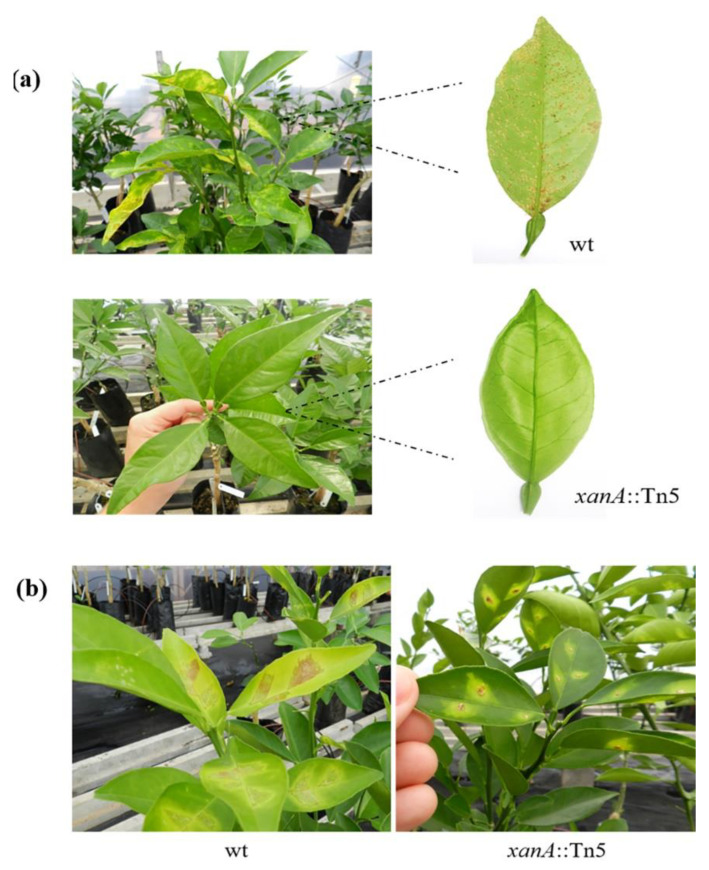
Pathogenicity assays. (**a**) Symptoms of sweet orange leave inoculated by spraying with wt and *xanA*::Tn5 mutant strains. (**b**) Symptoms of sweet orange leave infiltrated with wt and *xanA*::Tn5 mutant strains. Symptoms were analyzed for 30 days post-inoculation and pictures of leaves are representative of six independent replicates. wt, wild-type; *xanA*::Tn5, EZ-Tn5 insertion mutant.

Previous studies have verified that xanthan plays a role in *X. citri* pathogenesis in detached leaves [12], however, this is not a natural condition to understand the host-pathogen interaction. We then inoculated the wild-type strain and *xanA*::Tn5 on host leaves by two different methods: spray and infiltration. The symptoms evaluation was monitored for 30 days after inoculation. No symptoms were observed in *xanA*::Tn5 when inoculated by spray, in opposition to severe citrus canker symptoms caused by wt cells (Figure 2a). However, when the strains were inoculated by infiltration, *xanA*::Tn5 mutant was able to cause canker symptoms, even though less severe than those observed by wt strain (Figure 2b). These results indicate that *xanA* plays an important role in the epiphytic stage of the *X. citri* lifestyle, since *xanA* mutated allele affected this ability, thus the bacterium was not able to cause disease when inoculated by spray, a method that mimics a natural condition of *X. citri* infection.

### 3.3. xanA Plays a Critical Role in Controlling Important Features in X. citri

To verified that the Tn5 mutation did not have a polar effect, we generated a deletion mutant of the *xanA* gene in *X. citri* that was also complemented with the construct pUFR053-*xanA*. Both *xanA* mutants (*xanA*::Tn5 and Δ*xanA*) and wt strains were tested to determine their ability to form biofilm on the abiotic surface, sliding motility, gum production, and pathogenicity. The growth curve of the mutant strains was not different from that in the wild-type (Figure S1).

**Figure 3:**
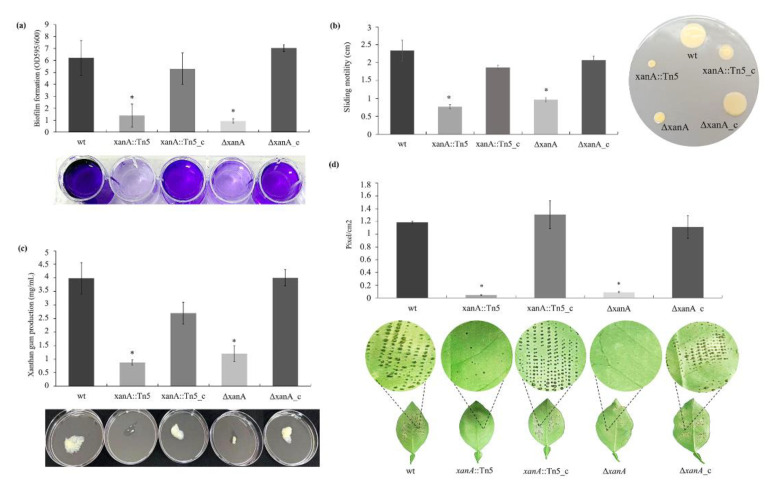
Biofilm, sliding motility, xanthan gum production, and pathogenicity of *X. citri* strains. (**a**) Biofilm formation on polystyrene surface. Values are expressed as the means ± standard deviations of six independent replicates. (**b**) Sliding motility phenotypes on SB agar medium plates after 48 h of incubation at 28 °C. Values are expressed as the means ± standard deviations of three independent replicates. (**c**) Xanthan gum production by *X. citri* strains. (**d**) Sweet orange leaves microneedle and inoculated with the different *X. citri* strains (10^8^ CFU/mL). Pictures are representative of five leaves for each strain. wt, wild-type; *xanA*::Tn5, transposon mutant; *xanA*::Tn5_c, complemented transposon mutant; Δ*xanA*, deletion mutant; Δ*xanA*_c, complemented deletion mutant. * indicates significant difference by t-test (*p* < 0.05) compared with wt strain.

Two independent mutant strains of *xanA* showed a decreased in biofilm formation, as shown in the first test (Figure 3a), and in sliding on medium plates (Figure 3b). On the plate surface, the diameters of the bacteria sliding resulted from migration away from the inoculation points in the agar surface were about 3-fold higher for wt than both *xanA* mutant strains after 48 h at 28 °C (Figure 3b). The complemented strain showed results like those of the wild-type strain, indicating that the sliding motility phenotype of the mutant was restored. Swimming motility analyses were also performed, but no difference was observed between the mutant and the wt strains (data not shown).

Xanthan gum amount also decreased about 4.5-fold in *xanA*::Tn5 and 3.4-fold in Δ*xanA* strain compared to the wt strain. This phenotype was restored to the wt level by complementation of the mutants, with no significant difference (*p* < 0.05) between *xanA*::tn5_c, Δ*xanA*_c, and wt, respectively. These differences in the amount of xanthan gum extracted from supernatants of these strains can also be visually observed on the plates (Figure 3c).

Impairment of pathogenesis after bacterial inoculation on leaf surfaces that has been verified for *xanA*::Tn5 mutant strain was also confirmed by Δ*xanA* and restored in *xanA* complementation strains (Figure 3d). A large number of pustules was observed on the leaves surfaces at 15 days after inoculation of wt and both complemented strains (*xanA*::Tn5_c and Δ*xanA*_c). However, very few pustules were observed on the leaves surfaces inoculated with each of the mutants (*xanA*::Tn5 and Δ*xanA*) strains.

### 3.4. Xanthan Gum from xanA Mutant Have Altered Monosaccharides Content

Xanthan gum produced by wt and *xanA*::Tn5 showed different percentages of some specific monosaccharides by HPLC analysis (Table 3). Xanthan gum produced by *xanA*::Tn5 exhibited a higher proportion of mannose, galactose, and xylose and reduced rates of rhamnose, glucuronic acid, and glucose. Thus, monosaccharide composition analysis indicated that *xanA* deletion altered the xanthan composition.

### 3.5. XanA Is Highly Conserved in the Xanthomonads Group

**Figure 4:**
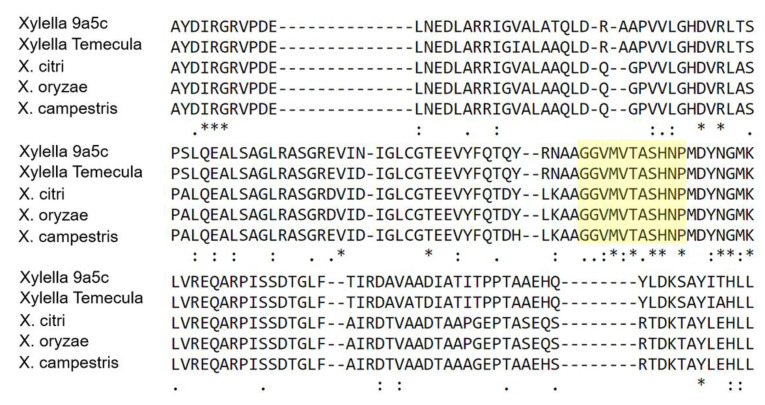
Sequence alignments of XanA homologs. The * indicates the presence of a conserved motif. The highlight region (GGVMVTASHP) characterizes the conserved domain Phosphoglucumutase and phosphomannomutase phosphoserine (PS00710). Abbreviations are as follows: Xylella 9a5c—Xylella fastidiosa 9a5c (XF_0260), Xylella Temecula—Xylella fastidiosa Temecula (PDO213), X. citri—X*anthomonas citri* subsp. *citri* (XAC3579), X. oryzae—*Xanthomonas oryzae* pv. *oryza*e (PXO_03174), XCC00626, X. campestris—*Xanthomonas campestris* pv. *campestris* str. ATCC 33913 (XCC0626). The protein motif was identified in Pfam (http://pfam.xfam.org/). Alignments were performed from residue 8 to residue 157 of *X. citri* XanA.

BLASTP search revealed that XAC3579 is highly conserved in other plant pathogenic species, including, *X. oryzae* pv. *oryzae* (99% identity), *X. campestris* pv. *campestris* (96% identity), and *Xylella fastidiosa* Temecula (84% identity) and *Xylella fastidiosa* 9a5c (84% identity). The phosphoglucomutase and phosphomannomutase, as well as XanA, share a conserved domain I that contains the catalytic phosphoserine (Figure 4), suggesting that this enzyme may be critical for cellular functions and give adaptative advantage for bacterial cells.

## 4. Discussion

In this work, we showed changes in the xanthan gum monosaccharide content that were associated with the *xanA* function in *Xanthomonas*. Disruption of *xanA* in *X. citri* genome, which codes for enzyme XanA that is necessary for isomerization of glucose-6-phosphate to glucose-1-phosphate and conversion of mannose-6- phosphate to mannose-1-phosphate [12,26,27], led to impairment of xanthan gum production. PGM/PMM possibly works as a valve, rerouting the metabolic flux originating from hexose phosphates either toward the biosynthesis of lipopolysaccharides (LPS) or xanthan, or the generation of energy or building blocks, such as amino acids, for cellular growth [27,28]. Thus, with the lack of XanA synthesis, the PGM/PMM activity levels decreased and consequently, the conversion of the substrates to UDP-D-glucose and GDP-mannose was compromised, leading to a reduction of glucose and mannose activated monosaccharides. This explains the lower percentages of glucose and mannose in the xanthan composition in *xanA*::Tn5 mutant compared with that observed in the wild-type strain. Indeed, the *xanA* deletion resulted in a lack of both activated monosaccharides UDP-glucose and GDP-mannose, which led to the formation of a xanthan gum structurally different [29].

For completion of xanthan gum synthesis, different steps are involved, and they require specific substrates and specific enzymes during this biosynthetic process. If either the substrate or the enzyme is absent, the step is compromised [30]. Some authors have shown that when mutation of specific genes involved in the xanthan gum synthesis are made to simplify the repeating unit structure, the xanthan gum yield was much lower as than that produced from the wild-type strain [29]. Betlach et al. [31] constructed a mutant lacking the glucuronic acid residues and the pyruvate and as a result, the xanthan gum solution produced by this strain was highly viscous. As we showed here, mutation of *xanA* reflected in changes of the repeating unit in the xanthan structure, and consequently, the xanthan gum yield was much lower than that produced from the wild-type and complemented strains, although low levels of xanthan gum remained detectable. The remaining xanthan may be explained by the presence of the *xanB* gene in the *X. citri* genome which codes for the enzyme phosphomannose-isomerase-GDP-mannose pyrophosphorylase that has involvement in GDP-mannose synthesis, one of those xanthan precursors [11].

The drastic reduction of xanthan gum in *xanA* mutants (*xanA*::Tn5 and Δ*xanA*) was responsible for the decrease of biofilm formation, sliding motility, and pathogenicity observed in this work. As xanthan gum contributes to the bacterial attachment to surfaces [8] and biofilm development with an organized structure on both abiotic and biotic surfaces [4], we suggest that such features are associates with the *xanA* function in determining EPS content. These findings, together with the demonstration that biofilm formation in *xanA* mutants can be complemented by the pUFR53-*xanA* construct, strongly suggest that a structurally intact xanthan gum harboring a particular composition of monosaccharides is critical for the *X. citri* lifestyle. Therefore, xanthan gum can affect bacterial cell adherence on host tissue and survival under stress conditions, as had already been demonstrated by other authors [32].

Further, sliding motility was previously shown to be promoted by xanthan gum and inhibited by type IV pili in *X. citri* [3,23]. These previous results corroborate our data which shows that *xanA* mutants also had a significant reduction in sliding motility compared with the wild-type strain. Consistently, it has been proposed that xanthan acts as a surfactant or surface-wetting agent to facilitate this type of movement [33,34]. Indeed, *xanA*::Tn5 and Δ*xanA* displayed less spread on the medium surface that may be due to low levels of the xanthan produced by these mutant strains, since anterior works have shown that *X. citri* mutants impaired in xanthan gum production also have sliding motility altered [3,4,5,35,36].

Moreover, reduced xanthan gum has also been related to the impairment of *X. citri* pathogenesis [3,12,13]. In our study, likewise, we also observed fewer symptoms when the *xanA*::Tn5 mutant cells are infiltrated into the leaves. Since inoculation by infiltration breaks the first physical defense layer, *X. citri* can use subsequent pathogenicity mechanisms, such as the type III secretion system to inject specific effectors into host cells, culminating in the development of citrus canker symptoms. Accordingly, previous results showed that the *xanA* mutant retained its infectivity when infiltrated into leaves and the authors speculated that PGM itself is not critical to canker progression, but it is related to the inoculation method used, which does not simulate the natural process of infection by *X. citri* [12]. Even though infiltration does not reflect a natural infection condition, *xanA* has a role when the bacteria are in plant mesophyll. On the other hand, we show that *xanA*::Tn5 caused no symptoms in sweet orange leaves after spraying, a method that simulates the natural infection condition. These results suggest that *xanA* has an important role in the epiphytical stage on the leaves that is essential for the interaction with the host, including adaptive advantage for bacteria cell survival under stress conditions and invasion of parenchyma tissue, which is required for the pathogenesis of *X. citri*.

Our data expanded the investigation on the *xanA* function, showing its role in affecting the monosaccharide composition of xanthan gum. The modified xanthan produced by *xanA* mutant compromised important features in *X. citri* and strongly impaired its ability to cause disease when in the epiphytic stage. Thus, chemical compounds that target the metabolic pathways involved in the activation of monosaccharides such as UDP-D-glucose and GDP-mannose, which was shown in this work that interferes in pathogenicity, could be a strategy to impair citrus canker disease. Further, as *xanA* is highly conserved in many bacteria, this strategy could be applied to other plant pathogenic species.

## Figures and Tables

**Table 1:** Primers used to transposon insertion point confirmation.

Primer	Nucleotide Sequence (5′–3′)
KanA	CATGCAAGCTTCAGGGTTGA
KanB	GCGGGGATCCTCTAGAGTCG
KanC	ACCTACAACAAAGCTCTCATCAACC
AD1	NTCGA(G/C)T(A/T)T(G/C)G(A/T)GTT
XAC3579F	TATTTCCAGACCGATTACCTCA
XAC3579R	GAAACTCCAAAGTGCGTCTATG

**Table 2:** Strains and plasmids used in this study.

Strains/Plasmids	Characteristics	Reference or Source
*Escherichia coli*	F- ɸ80d*lacZ*hM15 h (*lacZ*YA-*arg*F) U169 endA1 deoR *recA*1 hsdR17(rK- mK+) *phoA* supE44 λ- thi-1 *gyrA*96 relA1	[17]
*Xanthomonas citri* subsp. *citri* (*X. citri*)	306 Syn. *X. axonopodis* pv. *citri* strain 306; wild-type, Rf^r^, Ap^r^	[16,18]
A7*/xanA*::Tn5 (*xanA*^-^)	*xanA* (XAC3579), Km^r^. Transposon (Tn5) mutant.	[14]
*xanA*::Tn5_c (*xanA*^+^)	*xanA* (contained in puFR053, Gm^r^)	This study
Δ*xanA* (*xanA*^-^)	Deletion (Tn5) mutant; *xanA* (XAC3579), Ap^r^.	This study
Δ*xanA_c* (*xanA*^+^)	*xanA* (contained in puFR053, Gm^r^)	This study
pNPTS138	pNPTS138, Kmr, *sacB*, lacZα+	M. R. Alley, unpublished
pNPTS_*xanA*	*xanA* gene cloning on pNPTS138	This study
pUFR053	IncW Mob+mob (P) lacZα+ Par+, Cmr, Gmr, Km^r^, shuttle vector	[19]
pUFR053_*xanA*	*xanA* (XAC3579) gene cloning on pUFR053	This study

Ap_r_, Km_r_, Gm_r,_ and Rf_r_ indicate resistance to ampicillin, kanamycin, gentamicin, and rifamycin, respectively.

**Table 3:** Percentages (%) of monosaccharides present in the xanthan gum produced by *Xanthomonas citri* subsp. *citri* strains.

*X. citri* Strains	%						
	Man	Rha	GlcA	GalA	Glc	Gal	Xyl
wt	51.86	6.20	3.45	0.39	27.32	4.60	1.92
*xanA*::Tn5	58.95	1.58	0.48	0.35	16.68	7.98	2.64

Man (mannose), Rha (ramnose), GlcA (glucuronic acid), GalA (galacturonic acyd), Glc (glucose), Gal (galactose) and Xyl (xylose).

## Supplementary Materials

The following are available online at https://www.mdpi.com/article/10.3390/microorganisms9061176/s1, Figure S1. Growth curve of X. citri strains in NBY medium.

## References

[1] Graham J.H., Gottwald T.R., Cubero J., Achor D.S. (2004). *Xanthomonas axonopodis* pv. *citri*: Factors affecting successful eradication of citrus canker. Mol. Plant Pathol..

[2] Gottwald T.R., Pierce F., Graham J.H. (2002). Citrus Canker: The Pathogen and Its Impact Plant Health Progress Plant Health Progress. Plant Health Prog..

[3] Granato L.M., Picchi S.C., Andrade M.O., Takita M.A., de Souza A.A., Wang N., Machado M.A. (2016). The ATP-dependent RNA helicase HrpB plays an important role in motility and biofilm formation in *Xanthomonas citri* subsp. *citri*. BMC Microbiol..

[4] Rigano L.A., Siciliano F., Enrique R., Sendín L., Filippone P., Torres P.S., Qüesta J., Dow J.M., Castagnaro A.P., Vojnov A.A. (2007). Biofilm formation, epiphytic fitness, and canker development in *Xanthomonas axonopodis* pv. *citri*. Mol. Plant Microbe Interact..

[5] Granato L.M., Picchi S.C., De Oliveira Andrade M., Martins P.M.M., Takita M.A., Machado M.A., De Souza A.A. (2019). The EcnA antitoxin is important not only for human pathogens: Evidence of its role in the plant pathogen *Xanthomonas citri* subsp. *citri*. J. Bacteriol..

[6] Guo Y., Zhang Y., Li J.-L., Wang N. (2012). DSF-mediated quorum sensing plays a central role in coordinating gene expression of *Xanthomonas citri* subsp. *citri*. Mol. Plant Microbe Interact..

[7] Guo Y., Figueiredo F., Jones J., Wang N. (2011). HrpG and HrpX play global roles in coordinating different virulence traits of *Xanthomonas axonopodi*s pv. *citri*. Mol. Plant Microbe Interact..

[8] Li J., Wang N. (2011). The *wxacO* gene of *Xanthomonas citri* ssp. citri encodes a protein with a role in lipopolysaccharide biosynthesis, biofilm formation, stress tolerance and virulence. Mol. Plant Pathol..

[9] Malamud F., Conforte V.P., Rigano L.A., Castagnaro A.P., Marano M.R., Morais do Amaral A., Vojnov A.A. (2012). HrpM is involved in glucan biosynthesis, biofilm formation and pathogenicity in *Xanthomonas citri* ssp. *citri*. Mol. Plant Pathol..

[10] Jansson P.-E., Kenne L., Bengt L. (1975). Structure of the extracellular polysacchride from *Xanthomonas campestris*. Carbohydr. Res..

[11] Koplin R., Arnold W., Hotte B., Simon R., Wang G., Puhler A. (1992). Genetics of xanthan production in *Xanthomonas campestris*: The *xanA* and *xanB* genes are involved in UDP-glucose and GDP-mannose biosynthesis. J. Bacteriol..

[12] Goto L.S., Vessoni Alexandrino A., Malvessi Pereira C., Silva Martins C., D’Muniz Pereira H., Brandão-Neto J., Marques Novo-Mansur M.T. (2016). Structural and functional characterization of the phosphoglucomutase from *Xanthomonas citri* subsp. *citri*. Biochim. Biophys. Acta Proteins Proteom..

[13] Hung C.-H., Wu H.-C., Tseng Y.-H. (2002). Mutation in the *Xanthomonas campestris xanA* gene required for synthesis of xanthan and lipopolysaccharide drastically reduces the efficiency of bacteriophage (phi)L7 adsorption. Biochem. Biophys. Res. Commun..

[14] Baptista J.C., Machado M.A., Homem R.A., Torres P.S., Vojnov A.A., do Amaral A.M. (2010). Mutation in the *xpsD* gene of *Xanthomonas axonopodis* pv. *citri* affects cellulose degradation and virulence. Genet. Mol. Biol..

[15] Liu Y.G., Whittier R.F. (1995). Thermal asymmetric interlaced PCR: Automatable amplification and sequencing of insert end fragments from P1 and YAC clones for chromosome walking. Genomics.

[16] da Silva A.C.R., Ferro J.A., Reinach F.C., Farah C.S., Furlan L.R., Quaggio R.B., Monteiro-Vitorello C.B., Van Sluys M.A., Almeida N.F., Alves L.M.C. (2002). Comparison of the genomes of two *Xanthomonas* pathogens with differing host specificities. Nature.

[17] Hanahan D. (1983). Studies on transformation of *Escherichia coli* with plasmids. J. Mol. Biol..

[18] Schaad N.W., Postnikova E., Lacy G.H., Sechler A., Agarkova I., Stromberg P.E., Stromberg V.K., Vidaver A.K. (2005). Reclassification of *Xanthomonas campestris* pv. *citri* (ex Hasse 1915) Dye 1978 forms A, B/C/D, and E as *X. smithii* subsp. citri (ex Hasse) sp. nov. nom. rev. comb. nov., *X. fuscans* subsp. *aurantifolii* (ex Gabriel 1989) sp. nov. nom. rev. comb. nov., and X. Syst. Appl. Microbiol..

[19] El Yacoubi B., Brunings A.M., Yuan Q., Shankar S., Gabriel D.W. (2007). In planta horizontal transfer of a major pathogenicity effector gene. Appl. Environ. Microbiol..

[20] Sambrook J., Fritsch E., Maniatis T. (2012). Molecular Cloning.

[21] Andrade M.O., Farah C.S., Wang N. (2014). The Post-transcriptional Regulator *rsmA/csrA* Activates T3SS by Stabilizing the 5′ UTR of hrpG, the Master Regulator of *hrp/hrc* Genes, in *Xanthomonas*. PLoS Pathog..

[22] Amaral A.M., Toledo C.P., Baptista J.C., Machado M.A. (2005). Transformation of *Xanthomonas axonopodis* pv. *citri* by Electroporation. Fitopatol. Bras..

[23] Souza D.P., Andrade M.O., Alvarez-Martinez C.E., Arantes G.M., Farah C.S., Salinas R.K. (2011). A component of the Xanthomonadaceae type IV secretion system combines a VirB7 motif with a N0 domain found in outer membrane transport proteins. PLoS Pathog..

[24] Guzzo C.R., Salinas R.K., Andrade M.O., Farah C.S. (2009). PILZ Protein Structure and Interactions with PILB and the FIMX EAL Domain: Implications for Control of Type IV Pilus Biogenesis. J. Mol. Biol..

[25] Fu D., O’Neill R. (1995). Monosaccharide composition analysis of oligosaccharides and glycoproteins by high-performance liquid chromatography. Anal. Biochem..

[26] Mitra S., Cui J., Robbins P.W., Samuelson J. (2010). A deeply divergent phosphoglucomutase (PGM) of *Giardia lamblia* has both PGM and phosphomannomutase activities. Glycobiology.

[27] Zandonadi F.S., Ferreira S.P., Alexandrino A.V., Carnielli C.M., Artier J., Barcelos M.P., Nicolela N.C.S., Prieto E.L., Goto L.S., Belasque J. (2020). Periplasm-enriched fractions from *Xanthomonas citri* subsp. *citri* type A and *X. fuscans* subsp. *aurantifolii* type B present distinct proteomic profiles under in vitro pathogenicity induction. PLoS ONE.

[28] Schatschneider S., Huber C., Neuweger H., Watt T.F., Pühler A., Eisenreich W., Wittmann C., Niehaus K., Vorhölter F.J. (2014). Metabolic flux pattern of glucose utilization by *Xanthomonas campestris* pv. *campestris*: Prevalent role of the Entner-Doudoroff pathway and minor fluxes through the pentose phosphate pathway and glycolysis. Mol. Biosyst..

[29] Rosalam S., England R. (2006). Review of xanthan gum production from unmodified starches by *Xanthomonas campestris* sp.. Enzyme Microb. Technol..

[30] Becker A., Katzen F., Pühler A., Ielpi L. (1998). Xanthan gum biosynthesis and application: A biochemical /genetic perspective. Appl. Microbiol. Biotechnol..

[31] Betlach M., Capage D., Doherty D., Hassler R., Henderson N., Vanderslice R., Marrelli J., Ward M., Yalpani M. (1987). Genetically engineered polymers: Manipulation of xanthan gum biosynthesis. Industrial Polysaccharides Genetic Engineering, Structure/Property Relations and Applications.

[32] Li J., Wang N. (2014). Foliar application of biofilm formation-inhibiting compounds enhances control of citrus canker caused by *Xanthomonas citri* subsp. *citri*. Phytopathology.

[33] Murray T.S., Kazmierczak B.I. (2008). *Pseudomonas aeruginosa* exhibits sliding motility in the absence of type IV pili and flagella. J. Bacteriol..

[34] Malamud F., Torres P.S., Roeschlin R., Rigano L.A., Enrique R., Bonomi H.R., Castagnaro A.P., Marano M.R., Vojnov A.A. (2011). The *Xanthomonas axonopodis* pv. *citri* flagellum is required for mature biofilm and canker development. Microbiology.

[35] Guo Y., Sagaram U.S., Kim J., Wang N. (2010). Requirement of the *galU* gene for polysaccharide production by and pathogenicity and growth in planta of *Xanthomonas citri* subsp. *citri*. Appl. Environ. Microbiol..

[36] Malamud F., Homem R.A., Conforte V.P., Yaryura P.M., Castagnaro A.P., Marano M.R., Morais do Amaral A., Vojnov A.A. (2013). Identification and characterization of biofilm formation-defective mutants of *Xanthomonas citri* subsp. *citri*. Microbiology.

