# Supplementary material for: Modified Monosaccharides Content of Xanthan Gum Impairs Citrus Canker Disease by Affecting the Epiphytic Lifestyle of Xanthomonas citri subsp. citri
Source: Microorganisms. 2021 May 29;9(6):1176. doi: 10.3390/microorganisms9061176 (PMC8229982; doi:10.3390/microorganisms9061176)
Supplement: Supplementary file 1 [file microorganisms-09-01176-s001.zip › microorganisms-1187159-supplementary.pdf]

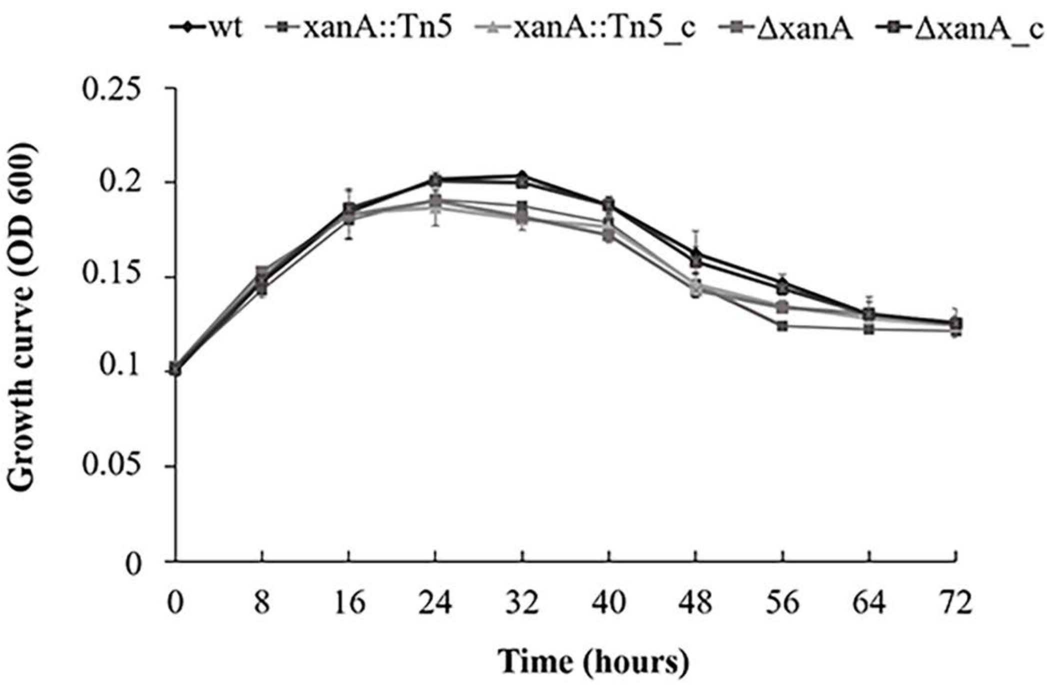

Figure S1. Growth curve of *X. citri* strains in NBY medium. Bacteria were grown at 28 °C in 96-well microtiter plates and measured in the Varioskan Flash Multimode Reader (Thermo Fisher Scientific) at 600 nm (OD600) for 72 hours. wt, wild-type; *xanA*::Tn5, transposon mutant; *xanA*::Tn5_c, complemented transposon mutant; Δ*xanA*, deletion mutant; Δ*xanA*_c, complemented deletion mutant.
